# A spreadsheet solution for off‐axis, noncentral enhanced dynamic wedge factors

**DOI:** 10.1120/jacmp.v4i3.2518

**Published:** 2003-06-01

**Authors:** B. D. Wichman

**Affiliations:** ^1^ Radiation Oncology Department Kansas City Cancer Centers 12200 W. 110th St. Overland Park Kansas 66210

**Keywords:** enhanced dynamic wedge, radiation therapy, off‐axis, wedge factor

## Abstract

Enhanced dynamic wedges can be very useful in increasing radiation therapy treatment efficiency. A drawback to their use is finding a factor analogous to a conventional wedge factor to calculate the dose to a point in a dynamically wedged field. A formalism has previously been published [J. Gibbons, Med. Phys. **25**, 1411–1418 (1998)] to determine this factor at the central point of a dynamically wedged field. This paper expands the use of the previous formalism to show efficacy at any point under a dynamically wedged field, provided the point is not in a penumbra region. Results of measurements taken with four clinically relevant field sizes, both symmetric and asymmetric, are given. These results show acceptable correlation between the original formalism and its expanded use to noncentral points.

PACS number(s): 87.53.–j, 87.66.–a

## INTRODUCTION

Enhanced dynamic wedges (EDWs) can be a valuable tool in the treatment of radiation therapy patients. They have many advantages over conventional, physical wedges. EDWs are easy to use, as they are computer driven, allow for more wedge angles, reduce peripheral dose, and do not require the therapist to enter the treatment room or lift heavy objects above the patient.

The main disadvantage of EDWs is their commissioning, entry of data into a treatment planning computer, rotation of the collimator to provide wedging in any direction, and providing a hand calculation of monitor unit settings for EDW fields. This project focuses on the latter.

Wedge factors for physical wedges are a fairly simple concept, easy to calculate and use in a hand calculation. EDW factors (EDWF) are more complex, though, because there is no instantaneous profile of a dynamically wedged field. Instead, the secondary collimator moves across the field, creating the wedging effect. A computer, based on “segmented treatment tables,” or STTs, tracks this movement. Several references give a full description of EDW properties^1–^, ^4^ and data gathering/commissioning.^5,^, ^6^

A methodology published a few years ago describes a mathematical solution to the problem of deriving EDW factors for symmetric and asymmetric fields.^7^ This paper gave results for both kinds of fields, showing close agreement between calculated and measured EDWF. What this paper did not discuss was EDW factors for points away from the geometric center of the field. Quite often for fields using EDWs there is an off‐axis, noncentral point of interest. Accurately knowing the EDW factor at this point is crucial to knowledge of the dose given at such a point.

## CALCULATION

The simplest method to calculate an EDWF is the monitor unit (MU) fraction method.^2,^, ^8–^, ^10^ This method approximates the EDWF as the fraction of MU a point is exposed to the radiation beam before the moving jaw blocks the point from primary beam exposure. This calculation can be done simply after analytical modelling of the “golden” STTs for dynamic wedges.^7,^, ^11^ A recently published method uses the MU fraction method coupled with correction factors based upon measured data to obtain center‐of‐field and off‐axis EDWF.^12^

Gibbons describes in detail an extension of the MU fraction model for determining the EDW factors.^7^ In essence, the EDW factor is not merely the ratio of the MU a point is exposed to over the total field MU, but also depends upon the field size in the *Y* collimator direction and greater amount of MU delivered in the latter parts of the collimator sweep through the field. This results in the following equations for EDWF at the geometric center of a field:^7,^, ^13^

(1) $$EDWF=\frac{W_{0}S_{G}(0)+W_{60}S_{G}(Y_{0})+W_{60}a_{1}b_{1}\alpha e^{\beta Y_{0}}\left(\frac{e^{(b_{-}){\bar{Y}}_{FJ-e}\left(b_{-}\right)Y_{0}}}{b_{-}}+e2b_{1}Y_{0}\frac{e^{-(b_{+}){\bar{Y}}_{FJ-e^{-(b_{+})Y_{0}}}}}{b_{+}}\right)}{W_{0}S_{G}(0)+W_{60}S_{G}({\bar{Y}}_{FJ})},$$

(2) $$W_{60}=\frac{\text{tan}(\theta )}{\text{tan}(60^{0})},$$

(3) $$W_{0}=1-W_{60},$$

(4) $$b_{\pm }\equiv b_{1}\pm \beta .$$

The golden STTs are calculated per the exponential function:

(5) $$S_{G}(Y)=a_{0}+a_{1}e^{b_{1}Y}.$$

Values for $a_{0}$, $a_{1}$, $b_{1}$, α, and β are given in Ref. 7 with respect to photon energy. The value θ is the EDW angle. The value $Y_{0}$ is the center point of the field. The value ${\bar{Y}}_{FJ}$ is the final moving jaw position, which is the fixed jaw position minus 0.5 cm (e.g., if the fixed jaw is at 5 cm, then ${\bar{Y}}_{FJ}=5-0.5=4.5$). The coordinate system is based upon the beam central axis being the origin with the positive axis in the direction of the fixed jaw.

While Gibbons' paper was concerned only with the EDW factor at the center of the field, this formalism can be applied to any off‐axis point if the field extends a distance past the calculation point. The third term in the numerator of Eq. (1) is a correction to the MU fraction model to account for the greater amount of MU given on the latter part of the collimator sweep for an EDW field. The term $Y_{0}$ in Eq. (1) can be replaced with $Y_{i}$, the point of interest (if $Y_{i}$ is in the center of the field, then $Y_{i}=Y_{0}$). If $Y_{i}$ is between $Y_{0}$ and the moving jaw's starting position, $Y_{s}$, the correction term will slightly underestimate the effect of the collimator sweep. This is because the correction term takes into account the fraction of dose delivered to the point of interest after the collimator has swept by. This fraction is greater if $Y_{i}$ is between $Y_{0}$ and $Y_{s}$ than if $Y_{i}$ is at the center of the field ($Y_{i}=Y_{0}$), which is assumed in Eq. (1). Conversely, if $Y_{i}$ is between $Y_{0}$ and $Y_{FJ}$, the correction term will slightly overestimate the effect of the collimator sweep. This effect will be greater for larger wedge angles, since the $W_{0}S_{G}(0)$ terms in Eq. (1) decrease with increasing wedge angle, finally going to zero at a wedge angle of 60°. This effect will also be greater with increased distance between $Y_{i}$ and $Y_{0}$. For this formalism, the field would need to extend a distance such that for an open field, the point of interest would not lie in the field penumbra.

This formalism is simple to input into a spreadsheet, so that the user inputs the EDW angle, the fixed jaw position, and the calculation point. The spreadsheet is then used to calculate the EDWF.

## METHODS AND MATERIALS

**Figure 1:**
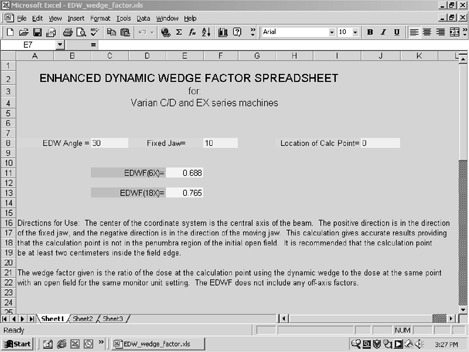
Spreadsheet for calculating EDWF.

Equations (1)–(5) were entered into a spreadsheet (Microsoft Excel®) for simple and quick calculation of the EDWF (Fig. 1). The user input consists of the EDW angle, the fixed jaw position, and the *Y* coordinate of the point of interest (in the coordinate system previously described). The spreadsheet continuously updates the EDWF for 6 and 18 MV as the information is entered.

Measurements were taken at three points each for four different field size/moving jaw configurations, for each EDW angle. The field sizes were based on typical clinical applications of EDW, with two fields being symmetric and two asymmetric. A point of interest was chosen in each direction from the center of the field. A measurement at the center of the field was also taken. The central point for the symmetric fields could be used to affirm the results of Gibbons' paper. A description of the fields used is found in Table I.

**Table I:** Description of fields and points of interest used for measurement of EDW factors.

Geometry	Field size (cm^2^)	Negative direction point of interest coordinate	Center of field coordinate	Positive direction point of interest coordinate	EDW orientation
*A*	5×5 symmetric	−1 cm	0 cm	+1 cm	OUT (Y1 fixed)
*B*	20×20 symmetric	−5 cm	0 cm	+ 5 cm	IN (Y2 fixed)
*C*	Y1=2, Y2=12, *=20	−8 cm	−5 cm	−1 cm	OUT (Y1 fixed)
*D*	Y1=0, Y2=20, *=10	−15 cm	−10 cm	−5 cm	OUT (Y1 fixed)

EDW factors for each setting measured were calculated and compared with factors from the equations. EDW factors (central or off‐axis) are determined as the ratio of a dose to a point of interest for a particular setup (field size and measurement depth) with EDW to the open field dose for the same setup.

Measurements were taken in a water phantom (Wellhofer “Blue Phantom”) with a 0.13 cm3 Wellhofer CC13 ionization chamber connected to a Standard Imaging CDX‐2000B electrometer. The chamber was centered at the point of measurement, long axis horizontal and perpendicular to the direction of jaw movement.

Measurements were taken on a Varian Clinac 2100EX for 6 and 18 MV. Depths of measurement for each energy were determined based upon typical clinical use of each energy. As such the depths chosen were 5 cm for the 6 MV beam and 10 cm for the 18 MV beam. The target‐detector distance was 100 cm for both energies.

It is important to emphasize that the EDWF found from Eq. (1) for an off axis, noncentric point does not include any off‐axis factors attributable to the open beam profile. When performing a manual monitor unit calculation on an off axis, noncentric point in an enhanced dynamic wedged field, a standard off‐axis factor for the point of interest should also be included.

## RESULTS

The tables below list the EDWF calculated using Eq. (1) as entered into the spreadsheet, the measured EDWF, and the percentage difference between the measured and calculated.

Tables II and III show results for measurement geometry *A*. For geometry *A*, a symmetric $5\times 5\;{\text{cm}}^{2}$ field, the results show excellent agreement for all EDW angles at each energy. The maximum difference between calculated and measured EDWF was <1% for both energies.

**Table II:** Calculated and measured EDWF for field geometry A, 6 MV photons.

	−1 cm			Center of field (0cm)			+ 1cm		
EDW angle	Calc.	Meas.	% diff.	Calc.	Meas.	% diff.	Calc.	Meas.	% diff.
10	0.971	0.972	0.10	0.980	0.986	0.61	0.989	0.994	0.51
15	0.956	0.958	0.21	0.970	0.973	0.31	0.984	0.986	0.20
20	0.941	0.941	0.00	0.959	0.961	0.21	0.978	0.982	0.41
25	0.926	0.931	0.54	0.948	0.953	0.53	0.973	0.977	0.41
30	0.909	0.907	−0.22	0.936	0.940	0.43	0.967	0.969	0.21
45	0.850	0.854	0.47	0.895	0.897	0.22	0.945	0.948	0.32
60	0.759	0.761	0.26	0.832	0.834	0.24	0.911	0.913	0.22

**Table III:** Calculated and measured EDWF for field geometry A, 18 MV photons.

	−1 cm			Center of field (0cm)			+1cm		
EDW angle	Calc.	Meas.	% diff.	Calc.	Meas.	% diff.	Calc.	Meas.	% diff.
10	0.977	0.978	0.10	0.985	0.984	−0.10	0.992	0.991	−0.10
15	0.967	0.968	0.10	0.977	0.977	0.00	0.988	0.989	0.10
20	0.955	0.958	0.31	0.969	0.969	0.00	0.984	0.982	−0.20
25	0.943	0.943	0.00	0.961	0.961	0.00	0.980	0.979	−0.10
30	0.930	0.931	0.11	0.952	0.953	0.11	0.975	0.973	−0.21
45	0.883	0.886	0.34	0.919	0.920	0.11	0.958	0.955	−0.31
60	0.810	0.810	0.00	0.868	0.866	−0.23	0.932	0.924	−0.86

Tables IV and V show results for measurement geometry *B*. For geometry *B*, a symmetric $20\times 20\;{\text{cm}}^{2}$ field, the results show excellent agreement at the lower EDW angles, with a generally increasing difference between calculated and measured values as EDW angle is increased. The maximum differences between calculated and measured EDWF were <2% for 6 MV and one and one half percent for 18 MV

**Table IV:** Calculated and measured EDWF for field geometry B, 6 MV photons.

	−5 cm			Center of field (0cm)			+5cm		
EDW angle	Calc.	Meas.	% diff.	Calc.	Meas.	% diff.	Calc.	Meas.	% diff.
10	0.843	0.846	0.36	0.877	0.876	−0.11	0.929	0.925	−0.43
15	0.777	0.779	0.26	0.825	0.823	−0.24	0.898	0.897	−0.11
20	0.715	0.716	0.14	0.777	0.775	−0.26	0.870	0.867	−0.34
25	0.657	0.657	0.00	0.731	0.728	−0.41	0.844	0.839	−0.59
30	0.602	0.601	−0.17	0.688	0.684	−0.58	0.819	0.813	−0.73
45	0.442	0.443	0.23	0.562	0.558	−0.71	0.746	0.740	−0.80
60	0.273	0.270	−1.10	0.430	0.424	−1.40	0.669	0.658	−1.64

**Table V:** Calculated and measured EDWF for field geometry B, 18 MV photons.

	−5 cm			Center of field (0 cm)			+5cm		
EDW angle	Calc.	Meas.	% diff.	Calc.	Meas.	% diff.	Calc.	Meas.	% diff.
10	0.885	0.886	0.11	0.914	0.915	0.11	0.952	0.952	0.00
15	0.834	0.833	−0.12	0.874	0.872	−0.23	0.931	0.929	−0.21
20	0.784	0.786	0.26	0.837	0.835	−0.24	0.910	0.908	−0.22
25	0.735	0.734	−0.14	0.801	0.798	−0.37	0.890	0.887	−0.34
30	0.688	0.686	−0.29	0.765	0.763	−0.26	0.870	0.865	−0.57
45	0.540	0.538	−0.37	0.653	0.650	−0.46	0.808	0.802	−0.74
60	0.368	0.366	−0.54	0.523	0.520	−0.57	0.736	0.727	−1.22

Tables VI and VII show results for measurement geometry *C*. For geometry *C*, an asymmetric field approximating one used often in breast planning in our clinic, the results show excellent agreement for all EDW angles for the 18 MV beam. As with geometry *B*, though, the results for the 6 MV beam show good agreement between calculated and measured EDWF for lower EDW angles, with agreement worsening as the angle is increased. The maximum differences between calculated and measured EDWF were <3% for 6 MV, and <1% for 18 MV

**Table VI:** Calculated and measured EDWF for field geometry C, 6 MV photons.

	−1 cm			Center of field (–5 cm)			–8cm		
EDW angle	Calc.	Meas.	% diff.	Calc.	Meas.	% diff.	Calc.	Meas.	% diff.
10	0.976	0.973	−0.31	0.948	0.948	0.00	0.933	0.931	−0.21
15	0.964	0.961	−0.31	0.922	0.920	−0.22	0.899	0.896	−0.33
20	0.952	0.950	−0.21	0.895	0.893	−0.22	0.864	0.862	−0.23
25	0.939	0.934	−0.53	0.867	0.862	−0.58	0.827	0.823	−0.48
30	0.925	0.916	−0.97	0.836	0.831	−0.60	0.788	0.783	−0.63
45	0.874	0.860	−1.60	0.726	0.715	−1.52	0.645	0.637	−1.24
60	0.794	0.772	−2.77	0.552	0.536	−2.90	0.419	0.409	−2.39

**Table VII:** Calculated and measured EDWF for field geometry C, 18 MV photons.

	−1 cm			Center of field (–5 cm)			–8cm		
EDW angle	Calc.	Meas.	% diff.	Calc.	Meas.	% diff.	Calc.	Meas.	% diff.
10	0.981	0.981	0.00	0.959	0.958	−0.10	0.945	0.946	0.11
15	0.973	0.972	−0.10	0.938	0.938	0.00	0.916	0.915	−0.11
20	0.963	0.962	−0.10	0.916	0.914	−0.22	0.888	0.887	−0.11
25	0.953	0.952	−0.10	0.893	0.891	−0.22	0.857	0.855	−0.23
30	0.942	0.940	−0.21	0.868	0.868	0.00	0.824	0.824	0.00
45	0.902	0.897	−0.55	0.778	0.777	−0.13	0.704	0.703	−0.14
60	0.838	0.830	−0.95	0.632	0.629	−0.47	0.509	0.507	−0.39

Tables VIII and IX show results for measurement geometry *D*. For geometry *D*, a field at the limit of *Y*‐direction asymmetry for EDW fields, the results mirrored those of geometry *C*. Agreement between the calculated and measured EDWF was excellent for all EDW angles for the 18 MV beam. The results for the 6 MV beam show good agreement between calculated and measured EDWF for lower EDW angles, with agreement again worsening as the angle is increased. The maximum differences between calculated and measured EDWF were <2% for 6 MV, except for the 60° EDW, where the discrepancy was close to 3% at the center of the field. The maximum differences for 18 MV were <1.5%.

**Table VIII:** Calculated and measured EDWF for field geometry D, 6 MV photons.

	−5 cm			Center of field (–10 cm)			–15cm		
EDW angle	Calc.	Meas.	% diff.	Calc.	Meas.	% diff.	Calc.	Meas.	% diff.
10	0.967	0.965	−0.21	0.942	0.945	0.32	0.927	0.927	0.00
15	0.949	0.947	−0.21	0.913	0.913	0.00	0.889	0.888	−0.11
20	0.931	0.929	−0.21	0.881	0.880	−0.11	0.849	0.846	−0.35
25	0.912	0.907	−0.55	0.848	0.846	−0.24	0.806	0.804	−0.25
30	0.890	0.881	−1.01	0.811	0.805	−0.74	0.759	0.755	−0.53
45	0.808	0.795	−1.61	0.669	0.662	−1.05	0.577	0.573	−0.69
60	0.660	0.641	−2.88	0.414	0.402	−2.90	0.253	0.248	−1.98

**Table IX:** Calculated and measured EDWF for field geometry D, 18 MV photons.

	−5 cm			Center of field (–10 cm)			–8cm		
EDW angle	Calc.	Meas.	% diff.	Calc.	Meas.	% diff.	Calc.	Meas.	% diff.
10	0.973	0.972	−0.10	0.951	0.951	0.00	0.935	0.936	0.11
15	0.959	0.958	−0.10	0.926	0.923	−0.32	0.901	0.903	0.22
20	0.944	0.941	−0.32	0.899	0.897	−0.22	0.866	0.865	−0.12
25	0.928	0.926	−0.22	0.870	0.866	−0.46	0.827	0.826	−0.12
30	0.911	0.909	−0.22	0.838	0.834	−0.48	0.786	0.785	−0.13
45	0.844	0.842	−0.24	0.718	0.713	−0.70	0.626	0.626	0.00
60	0.725	0.720	−0.69	0.504	0.497	−1.39	0.341	0.340	−0.29

## CONCLUSIONS AND DISCUSSION

There is agreement with the central axis EDWF results for geometries *A* and *B* and with the results from Gibbons' paper. Gibbons showed differences between 0–1% for measured EDWF values versus values obtained from Eq. (1), generally agreeing with our results.

For center‐of‐field points in the asymmetric fields of geometries C and D, Eq. (1) was accurate to >3% for 6 MV, and >1.5% for 18 MV. The results show excellent agreement between measurements of off‐axis, noncentral EDW factors and spreadsheet calculated values for 18 MV photon beams and any EDW angle. For the 6 MV beams, agreement is within 1% for all geometries for the 10°, 15°, 20°, 25°, and 30° wedges. With the 45° and 60° wedges, larger differences exist in the asymmetric field geometries. These differences are not of a magnitude that would preclude the use of Eq. (1) for manual monitor unit calculations.

For 6 and 18 MV, and almost all geometries and wedge angles, the ratio of measured/calculated EDWF is higher for points of interest between $Y_{0}$ and $Y_{s}$ than for points of interest between $Y_{0}$ and $Y_{FJ}$. As previously explained, this is expected as the corrective term in the numerator of Eq. (1) either overestimates or underestimates the correction depending which side of the center of field the point of interest is on.

The data presented in this paper show that a simple spreadsheet method can be used to calculate an off‐axis, noncentral EDWF for 6 and 18 MV photon beams from Varian C‐series linear accelerators. This method could be used for other energies as well if the STT for the energy of interest is fit to an equation of the form of Eq. (5).
